# The Effect of Calcium Ions on the Electrophysiological Properties of Single ANO6 Channels

**DOI:** 10.32607/actanaturae.27338

**Published:** 2024

**Authors:** D. O. Kolesnikov, E. R. Grigorieva, M. A. Nomerovskaya, D. S. Reshetin, A. V. Shalygin, E.V. Kaznacheyeva

**Affiliations:** Institute of Cytology of the Russian Academy of Sciences, St. Petersburg, 194064 Russian Federation

**Keywords:** ANO6, TMEM16F, calcium-activated chloride channels, patch-clamp technique, recording currents through single channels

## Abstract

Proteins belonging to the anoctamin (ANO) family form calcium-activated chloride channels (CaCCs). The most unusual member of this family, ANO6 (TMEM16F), simultaneously exhibits the functions of calcium-dependent scramblase and the ion channel. ANO6 affects the plasma membrane dynamics and phosphatidylserine transport; it is also involved in programmed cell death. The properties of ANO6 channels remain the subject of debate. In this study, we investigated the effect of variations in the intracellular and extracellular concentrations of calcium ions on the electrophysiological properties of endogenous ANO6 channels by recording single ANO6 channels. It has been demonstrated that (1) a high calcium concentration in an extracellular solution increases the activity of endogenous ANO6 channels, (2) the permeability of endogenous ANO6 channels for chloride ions is independent of the extracellular concentration of calcium ions, (3) that an increase in the intracellular calcium concentration leads to the activation of endogenous ANO6 channels with double amplitude, and (4) that the kinetics of the channel depend on the plasma membrane potential rather than the intracellular concentration of calcium ions. Our findings give grounds for proposing new mechanisms for the regulation of the ANO6 channel activity by calcium ions both at the inner and outer sides of the membrane.

## INTRODUCTION

Calcium-activated chloride channels are involved in the regulation of critical intracellular processes related to chloride transport and cellular membrane dynamics.

One of the isoforms of anoctamin, ANO6 (TMEM16F), simultaneously exhibits the functions of scramblase and the ion channel. Impaired functioning of ANO6 causes pathologic formation of the skeleton and placenta, miscarriage, cancer, and bleeding [1, 2, 3, 4]. Reduction of ANO6 activity is an effective approach to treating inflammatory respiratory diseases [5, 6].

Most of the studies focusing on channel functions have concerned ANO6 overexpression, which significantly alters the state of the cellular membrane, thus affecting channel properties. Furthermore, electrophysiological properties such as the open state lifetime of the channel, its single amplitude, and conductance can be adequately assessed only by recording the current flowing through single ion channels. The lack of these data obscures our understanding of the principles of ion channel functioning.

Being calcium-activated (EC_50_ = 10 µM at +40 mV), the ANO6 channel is not only regulated by intracellular calcium ions but can also conduct Ca^2+^ ions [4]. Thus, anionic conductance has been detected in some studies, while others have demonstrated that the channel has cationic conductance [4, 7]. Data on the effect of the extracellular calcium concentration on the electrophysiological properties of endogenous ANO6 channels is quite scarce. Furthermore, it is unclear how changes in the intracellular concentration of free calcium ions ([Ca^2+^]i) or membrane potential affect the kinetics and substates of single endogenous ANO6 channels.

This work aims to shed light on the dependence between the electrophysiological properties of single endogenous ANO6 channels and two major activitymodulating factors: the calcium concentration and potential.

## MATERIALS AND METHODS

**Cell culture**

The work was conducted using a HEK293T cell line from the collection of the Institute of Cytology, Russian Academy of Sciences (St. Petersburg, Russia). The cells were cultured in a liquid Dulbecco’s modified Eagle medium (DMEM) (PanEco, Russia) supplemented with 10% fetal bovine serum, 1% penicillin, 1% streptomycin, and 1% L-glutamine. The cells were re-inoculated onto coverslip fragments 16–48 h before the experiments.

**Reagents**

The reagents used in electrophysiological experiments were purchased from Sigma Aldrich (USA).

**Electrophysiological measurements of the current**

The currents flowing through single channels were recorded by the patch-clamp technique in the inside- out configuration using an Axopatch 200B (Axon Instruments, USA). The data were digitized using a Digidata 1322A analog-to-digital converter (Axon Instruments) with a discretization frequency of 5 kHz. The signal was passed through a built-in low-frequency (2 kHz) Bessel filter. In order to analyze the amplitude and the open state probability, as well as present the data, the recordings were additionally filtered at 110 Hz. No additional filtering was needed when analyzing the open-state lifetime of the channels; events shorter than 0.5 ms were not taken into account.

The composition of the extracellular solution (recording pipette solution) was as follows:

1) 105 mM CaCl_2_, 10 mM Tris-HCl, pH 7.4;

2) 1.5 mM CaCl_2_, 126 mM NaCl, 10 mM TeaCl, 10 mM glucose, 10 mM Tris-HCl, pH 7.4; and

3) 140 mM NaCl, 5 mM EGTA-Na, 10 mM Tris-HCl, pH 7.4.

The free calcium concentration [Ca^2+^]i was calculated using the Max Chelator software (Stanford University, USA). An intracellular solution with the calculated concentration of free calcium ions [Ca^2+^]i = 100 nM contained 130 mM CsGlutamate, 3.3 mM CaCl_2_, 5 mM MgCl_2_, 1 mM MgATP, 10 mM EGTA, and 10 mM HEPES. pH 7.2.

Solutions with calculated concentrations of free calcium ions of 0.2, 1 and 10 µM were obtained by adding CaCl_2_ at concentrations of 5, 8.5, and 9.82 mM, respectively, to the initial solution. The experiments were conducted at room temperature. The glass micropipettes had a resistance of 7–15 mΩ.

Channel activity was quantified using the (NPo) value, where N is the number of channels and Po is the open channel probability. NPo = (I)/i, where (I) is the average current through a membrane fragment; i is the amplitude of the open channel current. Since channel activity significantly varied over time, the average NPomax30 value (i.e., the average NPo measured during a 30s interval when channel activity was maximal) was used for our analysis.

**Statistical analysis**

The data were analyzed using the OriginPro2018 (Originlab, USA) and Clampfit 10.3 (Molecular Devices, USA) software. Data normality was tested using the Shapiro–Wilk test; the homogeneity of variance was assessed using Levene’s test for the equity of variances. Outliers, if any, were detected using the Grubbs’ test and expunged from the dataset. The frequencies of observation of CaCC were compared using the Fisher’s exact test. The open state probabilities for independent and constrained data were compared using the Student’s test and paired Student’s test, respectively. Multiple comparisons were conducted by ANOVA, with the Bonferroni correction. The data are presented as the Mean ± Standard error of the mean. Differences were considered significant at *p* < 0.05.

## RESULTS AND DISCUSSION

**Increasing activity of endogenous CaCCs ANO6 at higher extracellular concentrations of calcium**

**Fig. 1:**
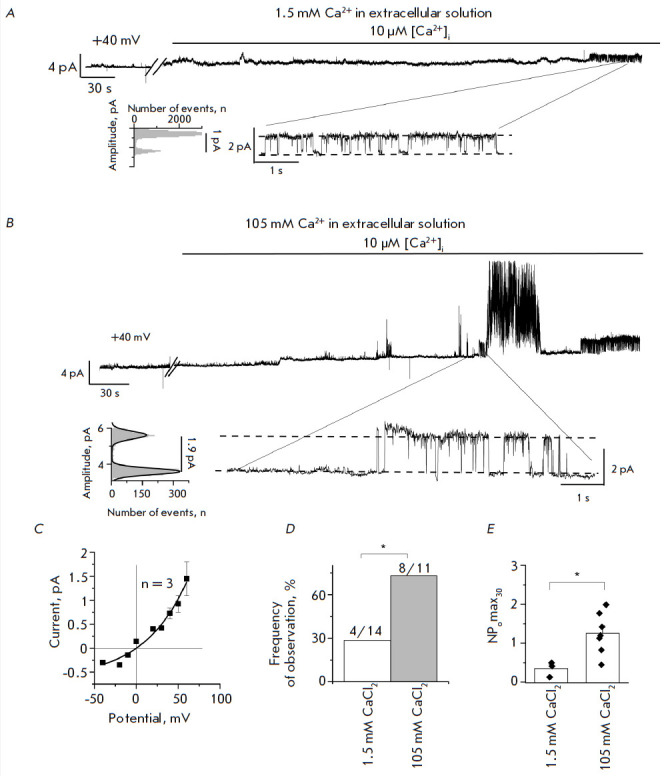
Activity of endogenous ANO6 CaCCs induced by application of 10 μM [Ca^2+^]i to the intracellular side of the plasma membrane in the inside-out configuration in the presence of 1.5 mM or 105 mM Ca^2+^ in the extracellular solution. Representative fragments of the recordings are shown, with expanded current traces and corresponding amplitude histograms at the bottom. (*A*) A fragment of the current recording through endogenous ANO6 CaCCs activated by application of 10 μM [Ca^2+^]i with 1.5 mM Ca^2+^ in the extracellular solution. (*B*) A fragment of the current recording through endogenous ANO6 CaCCs activated by application of 10 μM [Ca^2+^]i with 105 mM Ca^2+^ in the extracellular solution. (*C*) The average current–voltage relationship of endogenous ANO6 CaCCs in the presence of 1.5 mM Ca^2+^ in the extracellular solution. (*D*) The frequency of experiment observations with endogenous ANO6 CaCCs activated by application of 10 μM [Ca^2+^]i with 1.5 mM or 105 mM Ca^2+^ in the extracellular solution (*p* < 0.05). (*E*) NPomax30 of endogenous ANO6 CaCCs activated by application of 10 μM [Ca^2+^]i in the extracellular solutions of 1.5 mM and 105 mM Ca^2+^ (*p* < 0.05)

Previously, we had recorded and described single CaCC channels formed by ANO6 proteins [8, 9]. An extracellular solution containing 105 mM Ca^2+^ or 1.5 mM Ca^2+^ was used to study the effect of the extracellular calcium concentration on ANO6. Experiments were conducted in the inside-out configuration at a fixed membrane potential of +40 mV. Activation of endogenous ANO6 CaCCs was induced by adding a 10 µM [Ca^2+^]i solution to the intracellular side of the membrane (*Fig. 1 A,B*).

In the presence of a physiological concentration of CaCl_2_ (1.5 mM) in the extracellular solution, channel activation was observed in 28.5% of the experiments (4/14); the average NPomax30 was 0.35 ± 0.11 (*n* = 3) (*Fig. 1 A,C,D,E*). Meanwhile, in the presence of a high calcium concentration in the extracellular solution (105 mM CaCl_2_), channel activation was observed reliably more frequently: in 73% of the experiments with NPomax30 = 1.26 ± 0.2 (*n *= 7) (*p* < 0.05) (*Fig. 1 D,E*). Earlier, we demonstrated that a high concentration of calcium ions in a pipette solution could not activate endogenous ANO6 channels without additional stimulation by intracellular Ca^2+^ ions [8]. Therefore, the increase in channel activity correlates with a rising concentration of extracellular Ca^2+^.

Endogenous ANO6 channels are known both to be activated by calcium ions and to conduct them [4]. It can be assumed that at increased intracellular calcium concentrations, calcium ions permeate through a pore of the endogenous ANO6 channel, which can lead to the activity self-maintenance mode and further potentiation of channel activity.

Therefore, we demonstrated that both the open state probability and frequency of observation of endogenous ANO6 CaCCs in HEK293T cells increase with the concentration of calcium ions in the extracellular solution.

**The extracellular calcium concentration has no effect on the anionic conductance of channels**

The data on the anionic conductance of ANO6 are controversial. ANO6 has been described as anion- conducting [7] or cation-conducting [4] in different studies. The permeability of the channel for chloride ions depends on [Ca^2+^]i [10]. It was demonstrated that at low [Ca^2+^]i, the channel will preferably conduct cations rather than anions. The effect of the extracellular calcium concentration on anionic conductance remains poorly understood.

**Fig. 2:**
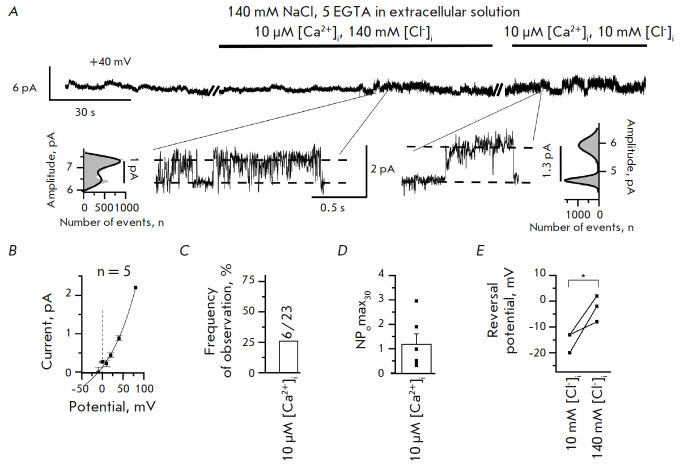
Activity of endogenous ANO6 CaCCs in the absence of divalent ions in the external solution. (*A*) A fragment of current recording through endogenous ANO6 CaCCs in an external solution containing 140 mM NaCl, 5 mM EGTA. Representative fragments of the recordings are shown, with expanded current traces and corresponding amplitude histograms at the bottom. (*B*) The average current–voltage relationship of endogenous CaCCs in an external solution containing 140 mM NaCl, 5 mM EGTA and intracellular solution based on CsGlutamate. (*C*) Frequency of observation of endogenous ANO6 CaCCs at 140 mM NaCl, 5 mM EGTA in an external solution. (*D*) The open state probability of endogenous ANO6 CaCCs at 140 mM NaCl, 5 mM EGTA in an external solution. (*E*) A shift in the reversal potential of endogenous CaCCs when one replaces an intracellular CsGlutamate solution with CsCl at 140 mM NaCl, 5 mM EGTA in the extracellular solution

In order to study this aspect, we removed all free calcium ions from the extracellular solution using the EGTA chelator. CaCC activation was induced by adding 10 µM [Ca^2+^]i to the intracellular side of the membrane (*Fig. 2A*). We observed activation of endogenous ANO6 CaCCs in the experiments (*Fig. 2B*).

In the presence of 140 mM NaCl, 5 mM EGTA in the extracellular solution, endogenous ANO6 CaCCs were observed in 26% of the experiments (6/23), the open-state probability being 1.17 ± 0.43 (*n *= 6). The activity of channels in the extracellular solution containing 140 mM NaCl, 5 mM EGTA showed no statistically significant differences compared to that in the presence of calcium at a physiological concentration (1.5 mM Ca^2+^) (*Fig. 1E*, *Fig. 2D*) (*p *> 0.05). These findings indicate that the physiological calcium concentration in the extracellular solution *per se *does not potentiate the activity of endogenous ANO6 CaCCs in HEK293T cells.

The variation in channel permeability for chloride ions can be assessed based on the changes in reversal potential as the Cl– concentration in the extracellular solution is varied. For this purpose, in the experiments with a calcium-free extracellular solution, we replaced the intracellular solution containing 130 CsGlutamate (10 mM [Cl–]) with a solution containing 130 CsCl (140 mM [Cl–]). When the intracellular solution containing 10 mM [Cl–] was replaced with that containing 140 mM [Cl–], the reversal potential shifted rightward by 12.7 ± 3.9 mV (*n *= 3) (*Fig. 2E*). Earlier, we demonstrated that the presence of 105 mM CaCl_2_ in the extracellular solution shifted the reversal potential rightward by 16 ± 2 mV when the intracellular solution was replaced in the same manner [8]. Hence, no statistically significant differences in the shift in the reversal potential were observed when using extracellular solutions containing or not containing calcium ions (*Fig. 2E*) [8].

Therefore, Ca^2+^ ions in extracellular solutions have no effect on the permeabilit

**An increase in [Ca^2+^]i leads to preferential activation of endogenous ANO6 channels with double amplitude**

Channels belonging to the anoctamin family of proteins are known to exist as homodimers. Each subunit contains a conducting pore. Inside each pore, there are two binding sites of calcium ions differing in their affinity [11].

It has been demonstrated that at low [Ca^2+^]i, when only one [Ca^2+^]i binding site is occupied, two pores of ANO1 channels function independently [12]. As [Ca^2+^]i increases, both binding sites of the [Ca^2+^]i ions in the pore are occupied, thus causing synchronous functioning of ion conduction pores in the channel [12]. However, it is currently unknown whether ANO6 also exhibits this property.

In order to answer this question, we recorded the activity of endogenous ANO6 channels in intracellular solutions with low (1 µM) and high (10 µM) [Ca^2+^]i in the inside-out configuration at a membrane potential of +40 mV. A pipette solution containing 105 mM CaCl_2_ was used to increase the frequency of observation of ANO6 CaCCs.

**Fig. 3:**
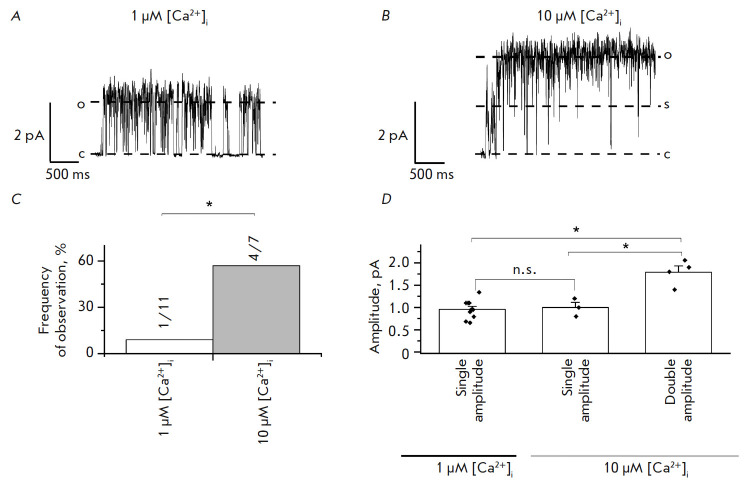
Amplitude of endogenous CaCC at 1 and 10 μM [Ca^2+^]i. The extracellular solution contained 105 mM CaCl_2_. The channels were activated by application of a solution with 1 or 10 μM [Ca^2+^]i. (*A*) A fragment of current recording through endogenous single-amplitude CaCC activated by application of 1 μM [Ca^2+^]i. c is the closed state of the channel; o is the open state of the channel. (*B*) A fragment of current recording through endogenous double amplitude CaCC activated by application of 10 μM [Ca^2+^]i. c is the closed state of the channel; o is the open state of the channel; and s is the substate. (*C*) The frequency of observation of endogenous CaCC ANO6 with double amplitude activated by 1 μM [Ca^2+^]i (white) or 10 μM [Ca^2+^]i (gray), *p* < 0.05. (*D*) The amplitude of endogenous ANO6 channels activated by 1 or 10 μM [Ca^2+^]i (*p* < 0.05)

We discovered that channels with amplitude 0.95 ± 0.06 pA (*n *= 10/11) were typically activated in the presence of 1 µM [Ca^2+^]i (*Fig. 3A*). When using the solution containing 10 µM [Ca^2+^]i, we observed activation of channels with a similar amplitude (1 ± 0.12 pA (1.79 ± 0.14 pA (*n *= 4/7)) (*Fig. 3B*). In the solution containing 1 µM [Ca^2+^]i, activation of channels with double amplitude was observed only in 9% of the experiments (*n* >= 1/11) (*p* < 0.05) (*Fig. 3 C,D*).

Hence, it can be inferred from our data that as [Ca^2+^]i increases, two conduction pores of endogenous ANO6 CaCCs start conducting current synchronously. Therefore, we can assume that the mechanisms of regulation by intracellular calcium ions are similar for ANO1 and ANO6 CaCCs.

**The open-state lifetime of the endogenous ANO6 CaCC depends on the membrane potential rather than on [Ca^2+^]i**

The current through an endogenous ANO6 CaCC is known to increase with the intracellular calcium concentration and membrane depolarization [10]. We have previously demonstrated that this is related to a higher open state probability of the channels, their amplitude, and conductance [8]. However, the increase in the current can also be related to changes in the open state lifetime of the channel.

In order to study this question, we analyzed the open state lifetime of endogenous ANO6 channels as a function of the [Ca^2+^]i concentration on the intracellular side of the membrane at a fixed membrane potential of +40 mV.

**Fig. 4:**
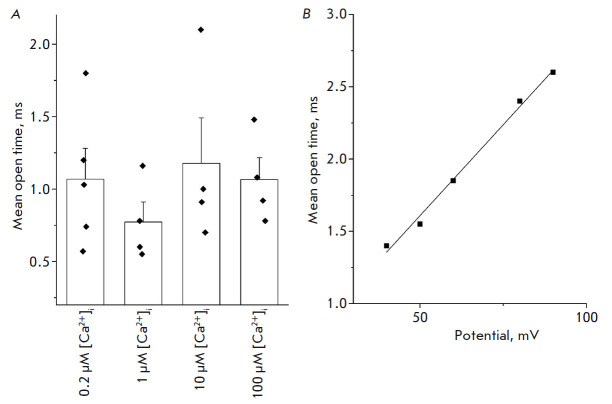
Effect of [Ca^2+^]i and the membrane potential on the open state lifetime of endogenous ANO6 channels in HEK293T cells. For the analysis, experiments were conducted in an inside-out configuration with 105 mM CaCl_2_ in the extracellular solution. (*A*) The average open state lifetime of endogenous CaCCs at +40 mV at various intracellular calcium concentrations (*p *> 0.05). (*B*) The dependence of the open state lifetime of endogenous CaCCs on the membrane potential at an intracellular calcium concentration of 100 μM (the data from a representative experiment are presented)

At 0.2 µM [Ca^2+^] i, the open state lifetime of channels was 1.07 ± 0.21 ms (*n *= 5); at 1 µM [Ca^2+^]i, – 0.77 ± 0.14 ms (*n *= 4); at 10 µM [Ca^2+^]i – 1.18 ± 0.31 ms (*n *= 4); and at 100 µM [Ca^2+^]i – 1.07 ± 0.15 ms (*n *= 4) (*p *> 0.05) (*Fig. 4A*). Therefore, the open state lifetime of channels was independent of variations in [Ca^2+^]i. An analysis of the dependence of the open state lifetime of endogenous ANO6 channels on the membrane potential at a fixed intracellular concentration of 100 µM [Ca^2+^]i demonstrated that the open state lifetime of CaCC increases linearly with increasing potential upon membrane depolarization (*Fig. 4B*).

Therefore, the increase in the current through endogenous CaCCs upon membrane depolarization is related not only to the higher conductance and open state probability of the channels (which was described earlier), but also to the longer open state lifetime of endogenous ANO6 channels. Meanwhile, variation in [Ca^2+^]i does not alter the open state lifetime of channels.

**Activity of ANO6 increases after transient switching to the negative membrane potential and back**

**Fig. 5:**
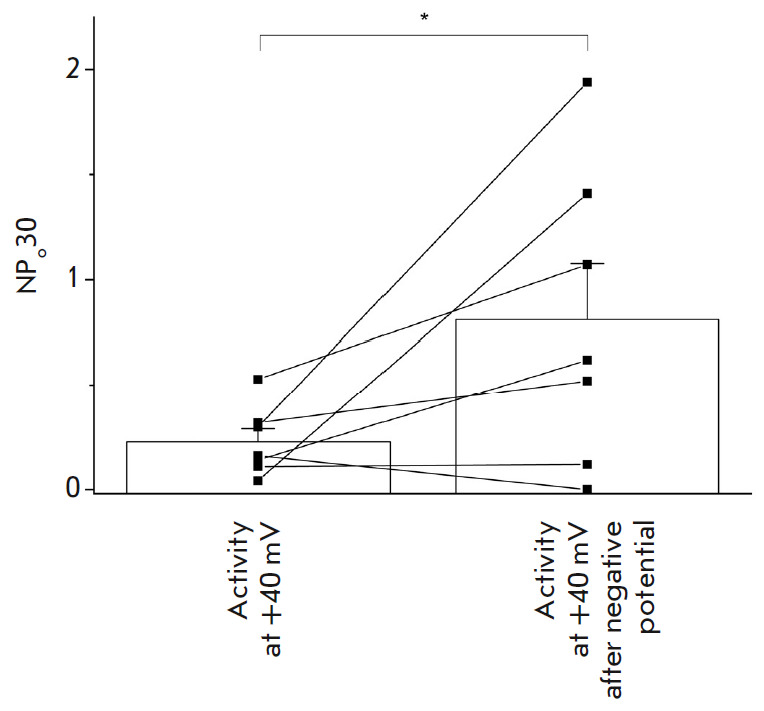
Activity of ANO6 channels at +40 mV before and after transient membrane potential switching to a negative value. The channels were pre-activated by adding 1 μM calcium to the intracellular side of the membrane. After the development of activity at +40 mV, the membrane potential was switched to negative and then returned to +40 mV

We have found that the activity of ANO6 channels at a potential of +40 mV increases after transient membrane potential switching to a negative potential and back to +40 mV. The experimental scheme is presented below: channels were activated by adding 1 µM [Ca^2+^]i to the intracellular side of the membrane at a potential of +40 mV; then the potential was maintained negative during 30 s, and a +40 mV voltage was reapplied. Potential switching back to +40 mV increased activity, on average from 0.23 ± 0.06 to 0.81 ± 0.26 (*n * >= 7, *p * < 0.05, *Fig. 5*). This effect was not observed at higher calcium concentrations (10 or 100 µM): presumably, the channels had been induced to the maximum extent and activity could not be increased any further.

It is known that there exists synergy between increasing the intracellular calcium concentration and plasma membrane depolarization in the activation of ANO6 channels. Thus, for overexpressed ANO6 channels, depolarization facilitates the interaction between calcium ions and intracellular binding sites, which increases channel activity [10]. In this study, we demonstrated for the first time that potential switching *per se* increases ANO6 activity. It is fair to believe that changes in the electric field at the instant when the membrane potential is switched from a negative value to +40 mV contributes to the conformational changes in endogenous ANO6 channels, which enable efficient binding of calcium ions in the channel pore vestibule. This regulation will presumably be particularly pronounced under physiological conditions upon membrane potential fluctuations (hyperpolarization followed by depolarization), since it theoretically reduces the calcium concentration needed to increase channel activity. It would be interesting to further study whether this effect depends on the rate of the switching from a negative potential to a positive one.

## CONCLUSIONS

By analyzing the kinetics of the functioning of single CaCCs, we have, for the first time, detected lengthening of the open state lifetime of the channel induced by an increase in membrane potential. Along with the previously reported increase in conductance and open state probability of the channels, this property seems to be responsible for the characteristic outward rectification of CaCCs. The intracellular calcium concentration had no effect on channel lifetime; however, an analysis of the channel amplitude demonstrated that increasing calcium concentration synchronizes functioning of the pores of the anoctamin dimer, thus increasing the current twofold.

Under experimental conditions, ANO6 channels are activated at high concentrations of intracellular calcium (several tens of micromoles), whereas the physiological calcium concentration in the cytoplasm is two orders of magnitude lower. We revealed that the cycle of membrane potential changes (hyperpolarization followed by depolarization) increases channel activity at a calcium ion concentration as low as 1 µM. This regulation can potentially also be observed under physiological conditions upon local membrane potential fluctuations.

High concentration of extracellular calcium ions increased channel activity, probably via nonselective entry of calcium ions and self-maintenance of channel activity. Since this effect was observed only in the presence of excessive calcium concentration (being almost two orders of magnitude higher than the physiological one), the physiological role of this process still needs to be elucidated. Channel permeability for chloride ions was independent of extracellular calcium concentration.

The identified mechanisms of regulation of ANO6 channel activity illustrate the potential pathways for fine-tuning of channel functioning under normal physiological and pathological conditions.

## Abbreviations

CaCC: calcium-activated chloride channels
ANO: anoctamins
[Ca2+]i: calculated intracellular concentration of free calcium ions
